# Supplementary material for: Chromothripsis during telomere crisis is independent of NHEJ, and consistent with a replicative origin
Source: Genome Res. 2019 May;29(5):737–49. doi: 10.1101/gr.240705.118 (PMC6499312; doi:10.1101/gr.240705.118)
Supplement: Supplemental Material [file supp_gr.240705.118_Supplemental_file_1.zip › contigs/annotated_contigs/DB84/contig.5.DB84_length_727_mean_cov_7.65061898212.docx]

**DB84_length_727_mean_cov_7.65061898212**

```
GAAATATTGCAGTGTACTAAAAGCAAACAAACAAATGAGGGTGTTTTGTCAATCGCATGTGTTGTGGAAATCTCATTCTCCCTCCAGAA
 >chr7:99146660-99146969 - E=4e-174 p=7e-03
TAATTCATGGATCTATGTAACATGAACATGTTTCAATATTCATAATACTGAATAATTATGCAGCTTAATTTCTTATTTTTAAGAAATAC

AAGTTCTCATTTTTTTCCCTATGCCCCAGTGGATCATCTTGTGTACCCTATGTACCCTACCTGATGGTTTTTTTTGTTTTTTGTTTTTT

GTTTTTTTTTTTTGAGACAGCGTCTTGCTCA|GTTGCCCAGGC|TGGAGTGCAGTGGCACTATCTTAGCTCACTGCAACCTCCTTCTCC
                                >chr17:8574960-8575146 - E=4e-100 p=0e+00
TGGGTTCAAGCGATTTTCCTGCCTCAGCCCCCAAGTAGCTGAGATTACAGGCACACACCACCACACCCGGCTAATTTTTGTATTTTTAG

ATGGGTTTTCACCACGTTGG|CCAGGCTGGTCTTGAACTCCT|GGGCTTAAACGATCCTGTCCCCCTCAGTCTCCCAAAGTTGCTAGGA
                     >chr1:231495792-231495876 - E=1e-38
TTACAGGATTG|AGCCAC|TTAGACGTGTGTAGGTATGCATATGTATACACACAAATTTTATATATATATATATATATATA|TATA|AT
            >chr1:231494830-231494902 - E=2e-31                                 >chr1
TTATTTATTTTTTGGAGACAGGGTCTTGCTCTGTCGCCCAGGCTGGAGTGCAGTGACGCGATCTCGGCTCACTGCAACCTCCACCTCGT
2:31774914-31775032 - E=4e-23
AGGTTCAAACAATTCTCGTGCCT
```
